# Combining legal epidemiology and implementation science to improve global access to medicines: challenges and opportunities

**DOI:** 10.3389/frhs.2023.1291183

**Published:** 2024-01-09

**Authors:** Jeff Lane, Andy Stergachis

**Affiliations:** ^1^ Department of Global Health, School of Public Health, University of Washington, Seattle, WA, United States; ^2^ Departments of Pharmacy and Global Health, Schools of Pharmacy and Public Health, University of Washington, Seattle, WA, United States

**Keywords:** law, policy, legal epidemiology, implementation science, medicines

## Abstract

Laws and policies affecting access to medicines have been in the global health spotlight for decades, yet our understanding of their effects remains substantially underdeveloped. The emerging field of legal epidemiology combined with the methods of implementation science presents an opportunity to help address this gap. Legal epidemiology refers to the scientific study and deployment of law as a factor in the cause, distribution, and prevention of disease and injury in a population. Legal epidemiology studies consist of a systematic collection and coding of laws and policies relating to a particular topic. Quasi-experimental or observational research methods can then be applied to take advantage of natural experiments resulting from heterogenous adoption and/or implementation of laws and policies. Often legal epidemiology studies fail to account for heterogenous law implementation processes, presenting a need and opportunity to integrate implementation science methods. Researchers may face challenges in integrating these methods for access to medicines studies, including data access issues and a complex legal and implementation environment. Yet, the opportunities presented by increasingly transparent legal environments, improved monitoring of medicine availability, universal health coverage expansion, and electronic health and insurance records integration may facilitate overcoming these challenges. Improved collaboration and communication between researchers, health authorities, manufacturers, and health providers from public and private sectors will be critical. In spite of the challenges, combining the fields of legal epidemiology and implementation science may present an important strategy toward creating a legal and policy environment that supports global and equitable access to medicines.

## Introduction

Laws and policies affecting access to medicines (i.e., drugs and vaccines) have been in the global health spotlight for decades, yet our understanding of their effects remains substantially underdeveloped. Inequitable and ineffective distribution of medicines grew on the global agenda in the 1990s and early 2000s in response to lack of universal access to medicines to treat HIV (1). Since then, objections have been raised about inequitable access to a wide range of medicines, including medicines for hepatitis C virus (2), pain management (3), cancer (4), COVID-19 (5), and pediatric pneumonia (6).

Debates around access to medicines laws and policies often focus on the role of intellectual property laws, in particular national patent laws. The widespread adoption of the Agreement on Trade Related Aspects of Intellectual Property Rights (TRIPS) in the mid-1990s led to near global adoption of national laws allowing for the patentability of medicines (7). TRIPS contained certain flexibilities that have been applied to improve access to medicines, but implementation of these flexibilities has been mixed (8). The ability of some countries to implement these flexibilities has also been limited by unilateral adoption of stricter patent laws or as a result of bilateral or multilateral trade agreements (7). Patent laws play an important role in the legal environment affecting access to medicines, especially for novel medicines. However, a range of laws and policies outside of patents also play important roles in affecting access to medicines, including those governing health insurance coverage, drug formularies and national essential medicines lists, medical products assessment and registration, import and export, taxes, medical product quality and safety surveillance, and licensing of health establishments and personnel (9).

Despite tremendous advancements in pharmaceutical development in recent decades, global access to medicines remains far from universal. Pharmacy supply chain, pricing, and affordability surveys have been implemented widely in resource limited settings and regularly find substantial stockouts and unaffordability (10). These surveys have used various methodologies, including the methodology developed by Health Action International and the World Health Organization (WHO) (10). A multi-country study of persons with chronic conditions in 2007-10 found that only 35% of respondents in Ghana, 33% of respondents in Kenya, 16% of respondents in Uganda, 49% of respondents in Jordan, and 38% of respondents in Philippines reported they had access to medicines to treat their chronic diseases (11). Attai, Khatib et al. measured the affordability of blood pressure lowering medicines in 20 countries and found the percentage of households unable to afford two blood pressure-lowering medicines was 31% in low-income countries, 9% in middle-income countries, and less than 1% in high-income countries (12). Other surveys have found substantial inequities in access to medicines, even in high income countries. For example, a 2023 survey in the U.S. found that 31% of respondents reported not taking their medicines as prescribed due to cost, with 21% reporting not filling their prescription or taking an over-the-counter medicine instead, and 12% reporting cutting pills in half or skipping a dose (13).

To help address this challenge, the Sustainable Development Goals included a target 3.b to provide access to affordable essential medicines and vaccines (14). Two indicators associated with this target directly call for monitoring the “Proportion of the target population covered by all vaccines included in their national programme” and the “Proportion of health facilities that have a core set of relevant essential medicines available and affordable on a sustainable basis.” Monitoring coverage of selected vaccines occurs regularly in most countries (15), but regular monitoring of progress against the indicator for access to non-vaccine medicines has been limited (16).

While we have growing evidence describing the problem of access to medicines globally, there have been relatively few real-world evaluations of the impact of laws and policies on access to medicines (17–19). The emerging field of legal epidemiology combined with the methods of implementation science presents an opportunity to help address this gap.

### Legal epidemiology

The field of legal epidemiology refers to the “scientific study and deployment of law as a factor in the cause, distribution, and prevention of disease and injury in a population.” (19) Legal epidemiology studies are grounded in a systematic collection and coding of laws relating to a particular topic, resulting in a database of laws that reveals meaningful differences between laws in different jurisdictions. These databases can be cross-sectional or longitudinal to show changes in laws by jurisdiction and over time. For example, the Policy Surveillance Program has published legal epidemiology databases coding laws in the U.S. on a wide range of public health topics, including health worker scopes of practice, health insurance coverage requirements, housing, environmental health, and food safety (20). The U.S. CDC has also developed and published databases mapping U.S. state laws affecting HIV/AIDS programs and services (21). Legal epidemiology studies can also include quasi-experimental or observational research methods that take advantage of natural experiments resulting from heterogenous adoption and/or implementation of laws (19). This natural heterogeneity is especially common in federalized systems that grant substantial lawmaking authority to local governments (e.g., states, provinces, counties, or cities). Under these natural experiments, quasi-experimental statistical analysis methods, such as interrupted time series or difference-in-difference estimation, can be used to explore causal inferences between law adoption, implementation, and public health outcomes (19).

While the emergence of the field of legal epidemiology is relatively new, the study of laws and their relationship to health has occurred for many decades (22). For example, MacKillop studied the effects of seatbelt legislation and reduction of highway speed limits in Ontario, Canada in the late 1970s (23). Rigotti and Pashos systematically mapped and coded anti-smoking laws in public spaces in U.S. cities and states (24), and Faden and Kass mapped U.S. state health insurance regulations for coverage of HIV/AIDS in the late 1980s (25).

Focus on the connection between law and health has grown following the establishment of the U.S. CDC Public Health Law Program in 2000 (26). Burris and colleagues at Temple University have been key leaders in the growth of the legal epidemiology field, including establishing the Public Health Law Research Program in 2009 (27). While the first use of the term legal epidemiology in journals indexed on Medline/PubMed did not occur until 2015 (28), between 2015 and August 2023, 89 articles indexed on Medline/PubMed have used the term legal epidemiology, showing the growing footprint of this field in the traditional public health and medical literature.^1^

Recent applications of legal epidemiology relating to laws affecting access to medicines include Salvant-Valentine, Carnes, et al. analysis of nurse practitioner prescribing laws on HIV pre-exposure prophylaxis prescriptions in the U.S. (29), and Aaltonen's analysis of the effect of austerity measures (which are essentially budget laws) on medication access in Finland (17). The potential application of legal epidemiology methods to the study international law was also recently explored by Poirier, Viens, et al. (30)

### Implementation science

Implementation science methods present an important complement to the emerging field of legal epidemiology because implementation science utilizes “methods to promote the adoption and integration of evidence-based practices, interventions, and policies into routine health care and public health settings to improve our impact on population health.” (31) The complexity of legal implementation can be especially important in access to medicines policy, because of the complex legal and operational systems that intersect with medicine procurement, distribution, prescribing, and dispensing. Often legal epidemiology studies focus on law adoption or effective dates, which fail to account for the sometimes lengthy and heterogenous process of implementing laws.

Implementation science has developed a range of implementation outcome frameworks well-suited to rigorously evaluate the implementation of laws affecting access to medicines. One example is Procter, Silmere, et al.'s taxonomy of implementation outcomes consisting of acceptability, adoption, appropriateness, feasibility, fidelity, implementation cost, penetration, and sustainability (32). These outcomes can be used to measure multiple attributes of law implementation (e.g., adoption vs. fidelity vs. penetration). Glasgow et al.'s Reach, Effectiveness, Adoption, Implementation, Maintenance/sustainment (RE-AIM) framework may also help with integrating implementation science outcomes into legal epidemiology analyses, especially to organize implementation outcomes data over time (33).

Other implementation science frameworks address laws and/or policies as a factor influencing implementation outcomes. A recent review by Crable, Lengnick-Hall, et al. found 26 implementation theories, models, or frameworks that address policy in some way (34). For example, the Aarons, Hulbert, Horwitz Conceptual Model of Evidence-Based Practice Implementation in Public Service Sectors (EPIS) includes a construct on service environment/policies (35). Crable, Lengnick-Hall, et al. recently made six recommendations to advance policy within EPIS and other dissemination and implementation frameworks (34). The Consolidated Framework for Implementation Research (CFIR) also includes a construct within its Outer Setting domain for Policies and Laws. CFIR defines Policies and Laws as “Legislation, regulations, professional group guidelines and recommendations, or accreditation standards support implementation and/or delivery of the innovation.” (36) However, as of November 2023, the CFIR website did not contain any guidance on quantitative or qualitative measures for coding policies and laws, illustrating the need to build out the law and policy construct within CFIR (37). While EPIS and CFIR each acknowledge the potential influence of laws and policies on implementation outcomes, legal epidemiology seeks to understand the effect of the laws themselves on public health. Therefore, integrating implementation science and legal epidemiology will require a greater recognition of law not just as a factor influencing intervention outcomes, but law as an intervention itself (34).

### Challenges & opportunities

Applying a combination of legal epidemiology and implementation science methods to study laws affecting access to medicines is not without challenges. Some of the most significant barriers involve lack of access to key data on laws, legal reforms, implementation processes, medicine availability, and medicine prices and affordability. Lack of collaboration between researchers, evaluators, implementers, and policymakers also presents barriers to integrating these fields. In spite of the challenges, we see many opportunities to vastly expand this work.

Conducting sound legal epidemiology requires access to the text of laws and regulations adopted in the jurisdictions that will be studied. Many local jurisdictions and even some countries lack a publicly available online version of existing and past legislation. However, governmental websites with current national laws and recently passed legislation are becoming more common (38). As more national and local legislatures and administrative agencies post their current and archived laws and policies online, conducting retrospective law and policy evaluations is becoming easier.

Implementation data relating to access to medicines can also be difficult to obtain at the population-level. In many countries, longitudinal data on medicine availability and stockouts do not exist or are typically not publicly available. As a result, a large percentage of medicine availability and affordability studies must collect primary data at a small number of facilities. However, ministries of health and others around the world have begun to collect and publish more data on medicine availability and distribution as a result of the COVID-19 pandemic response. For example, COVID-19 vaccine distribution dashboards were established in many countries (39). The WHO established a dashboard that aggregated COVID-19 vaccine distribution data globally (40), as did Johns Hopkins University (41). An increasing number of medicine regulatory authorities are also beginning to systematically monitor medicine shortages. Often these authorities publish notices of potential medicine shortages on public websites and databases (42, 43). Some countries, such as South Africa, have established medicine availability surveillance systems within departments of health to monitor medicine stock rates across the health system (44). These public medicine stock datasets and dashboards could be used to conduct legal epidemiology studies measuring the effect of law changes on medicine availability.

In many countries, medicines are dispensed through a mixed market of private and/or public pharmacies, which presents challenges in wholistically evaluating the effect of law reforms on stock and dispensing rates. The ongoing expansion of universal health coverage can help overcome this barrier (45), because insurance claim databases, where available, aggregate claims from public and private sector providers. These payor claims databases can include claims data from a single public insurance plan or can be structured as all payor claims databases that aggregate claims data across multiple public and private insurers (46). Ideally, we would also be able to assess the effects of medicines access-related law reforms on health outcomes, but integrating medicine access data with health outcomes data has been challenging in the absence of integrated record systems. However, health information exchanges and electronic medical record systems are becoming more prevalent in lower resource settings (47), allowing for the potential to integrate medicine prescribing, reimbursement, and dispensing data with health outcomes data.

In many countries, there continues to be a divide between researchers and implementers making it more challenging to access key data. However, formal academic-practice collaborations between departments of health and universities, sometimes referred to as academic health department partnerships, are becoming more common (48). These collaborations support mutually beneficial research and training collaborations between health departments and local universities. More than one hundred formal academic health department partnerships have been established in the U.S. (49), and similar academic-practice collaborations have been established in other countries, including Australia and Canada (50). Expanding the transdisciplinary nature of graduate training programs across medicine, pharmacy, law, public health, and public policy fields can help foster these types of collaborations (19).

Demand and funding for law and policy evaluation from policymakers and implementers can sometimes be lacking. Establishing formal policy research collaborations between policymakers and universities is helping to overcome this divide in some settings. Many legislatures have established formal legislative policy research units to conduct policy research and evaluation in support of legislation, such as the Parliamentary Research Service in Kenya (51) and the Congressional Research Service in the U.S (52). These units can support formative policy research to inform the development of new legislation or conduct retrospective evaluations of previously passed legislation. Policy research units can also be established within administrative agencies, such as a ministry or department of health or medicine regulatory authorities.

## Discussion

The emerging field of legal epidemiology may present an opportunity to advance the rigor and timeliness of evaluations of laws and policies affecting access to medicines. To realize this potential, however, we must ensure that the evaluation models address the important role of law and policy implementation. Implementation science outcomes and methods are well-positioned to support this goal. Researchers may face challenges in integrating these approaches, including data access issues and a complex legal and operational environment. Yet, the opportunities presented by increasingly transparent law and policy environments, improved medicine availability monitoring, universal health coverage expansion, and increasingly integrated electronic health and insurance record systems may overcome these challenges. Improved collaboration and communication between researchers, health authorities, pharmaceutical manufacturers, and pharmacies from the public and private sectors will be critical to this endeavor. In spite of the challenges, combining the fields of legal epidemiology and implementation science presents an important strategy in the path toward creating a legal and policy environment that finally achieves global and equitable access to medicines.

## Data Availability

The original contributions presented in the study are included in the article/Supplementary Material, further inquiries can be directed to the corresponding author.

## Footnotes

^1^Searches conducted on August 21, 2023.
